# ^1^H MR Spectroscopy in Gliomatosis: Is there a Sensitivity Issue?

**DOI:** 10.1155/2011/371073

**Published:** 2011-09-11

**Authors:** M. Szewczyk-Bieda, A. K. Kanodia, G. Main, M. S. Eljamel

**Affiliations:** ^1^Department of Clinical Radiology, Ninewells Hospital and Medical School, NHS Tayside, Dundee DDI 9SY, UK; ^2^Department of Neurosurgery, Ninewells Hospital and Medical School, NHS Tayside, Dundee DDI 9SY, UK

## Abstract

*Objective*. ^1^H MR spectroscopy (MRS) is widely performed for assessment of brain tumours and is considered a highly sensitive technique capable of differentiating benign from malignant conditions and tumour grading. *Method*. We present a case of a 69 year old woman who was suspected to have gliomatosis on MRI. *Results*. MRS performed using single voxel and chemical shift/multivoxel techniques was within normal limits. A repeat scan 6 months later showed progressive disease, and biopsy was performed that proved the diagnosis of glioblastoma. *Conclusion*. Normal MRS in a patient with suspicion of gliomatosis on MRI should not reassure clinicians into assuming a benign aetiology or a good prognosis in short term.

## 1. Introduction

MRS is widely performed as a research and diagnostic tool for assessment of brain tumours. Establishing a type and grade of intracranial tumour is often difficult with conventional magnetic resonance imaging (MRI), which is a principal imaging modality for its evaluation. MRS is a noninvasive method of acquiring information about metabolic activity of the brain tissue. It can be routinely obtained at the time of diagnostic MRI. 

There are several reports discussing the role of MRS in differentiating benign from malignant gliomas and radiation necrosis from tumour recurrence. This heavily relies on the high sensitivity of MRS in detecting metabolic abnormalities in brain tumours, particularly malignant ones. However, there is not much literature regarding negative predictive value or sensitivity of MRS in suspected brain tumours or gliomatosis cerebri.

We report a case of an intra-axial lesion, which had MRI appearances consistent with a low-grade diffuse glioma or gliomatosis, but MRS findings were entirely within limits for a normal brain tissue. A few months later, the histological analysis of the lesion, following biopsy, confirmed the diagnosis of glioblastoma multiforme (GBM).

## 2. Case Report

**Figure 1:**
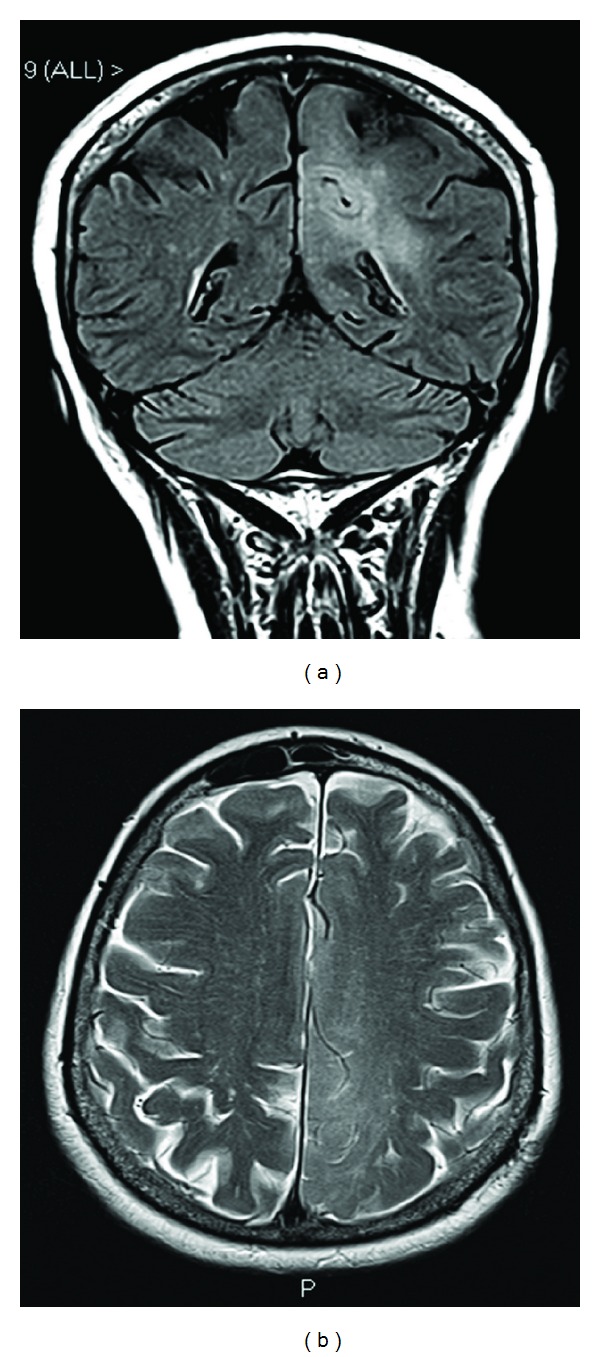
Coronal FLAIR (a) and axial T2 weighted images (b) showing extent of the lesion on MR scan taken at the time of MRS.

**Figure 2:**
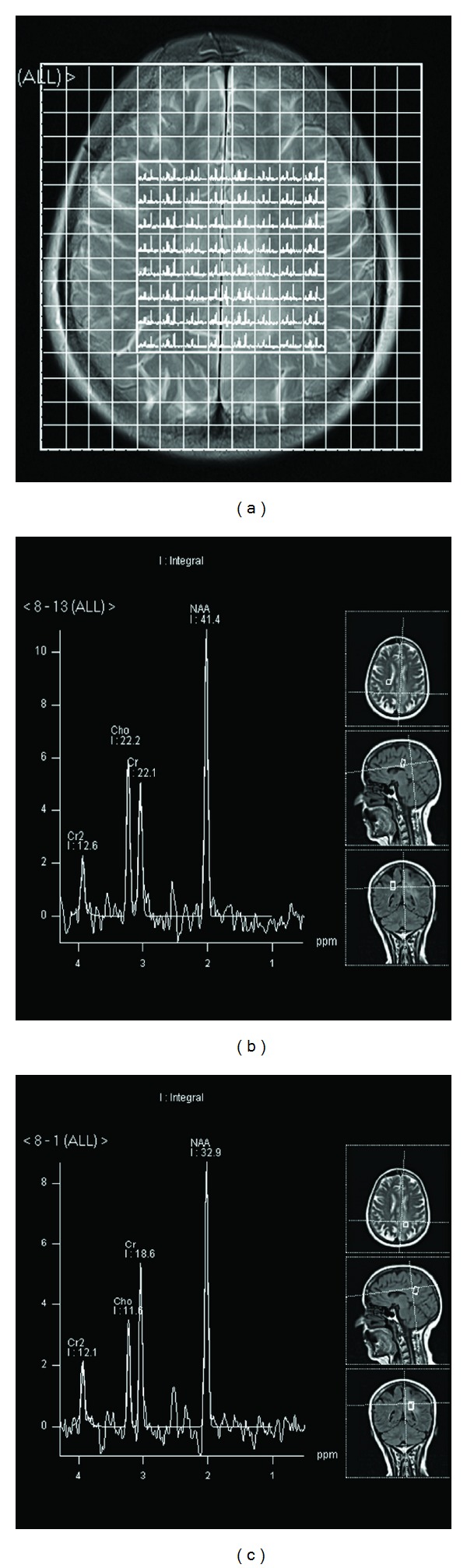
MRS CSI spectra (grid-2A) showing no alteration in metabolite ratios between normal (Figure 2(b)) and abnormal signal area of the brain (Figure 2(c)).

**Figure 3:**
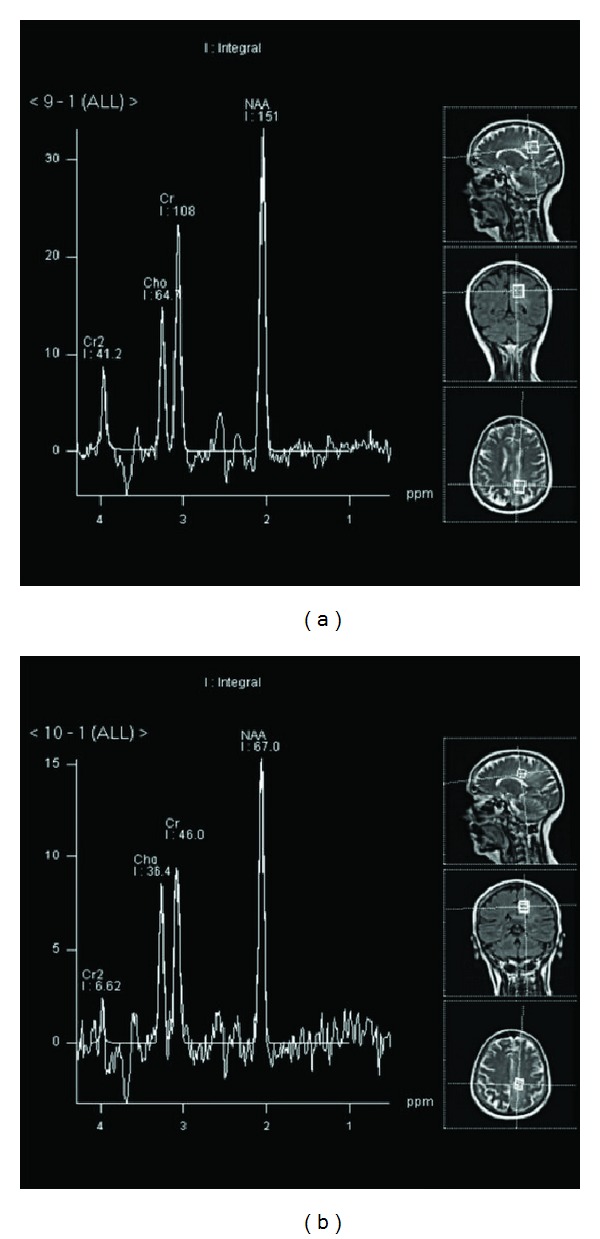
MRS SV spectrum from abnormal signal brain area (Figures 3(a) and 3(b)) showing NAA and Choline levels within normal limits.

A 69-year-old woman presented with a 14-month history of intermittent fornication in the distribution of the left mandibular (V3) division of the trigeminal nerve. MRI scan identified a presence of an ill-defined, diffuse lesion, involving cortex and subcortical white matter of the left occipital and parietal lobes extending into the frontal lobe, with only slight mass effect and relative preservation of underlying anatomy with no postcontrast enhancement. The MRI features were suggestive of low-grade diffuse glioma or gliomatosis cerebri. After five months time follow-up MR scan with MRS (1.5 T Siemens Avanto) using 2D CSI and single voxel (SV) technique (TE of 135 ms, TR of 1500 ms) was performed. The analysis of MRS findings was done using the standard software provided by the manufacturer. The MRI scan again confirmed the presence of extensive signal abnormality, which unchanged in size from the previous scan (Figure 1). The MRS (SV and CSI) through the abnormal areas was extensively assessed but showed no alteration in choline and N-acetyl aspartate (NAA) peaks compared to normal brain tissue and was entirely within normal limits (Figures 2 and 3).

**Figure 4:**
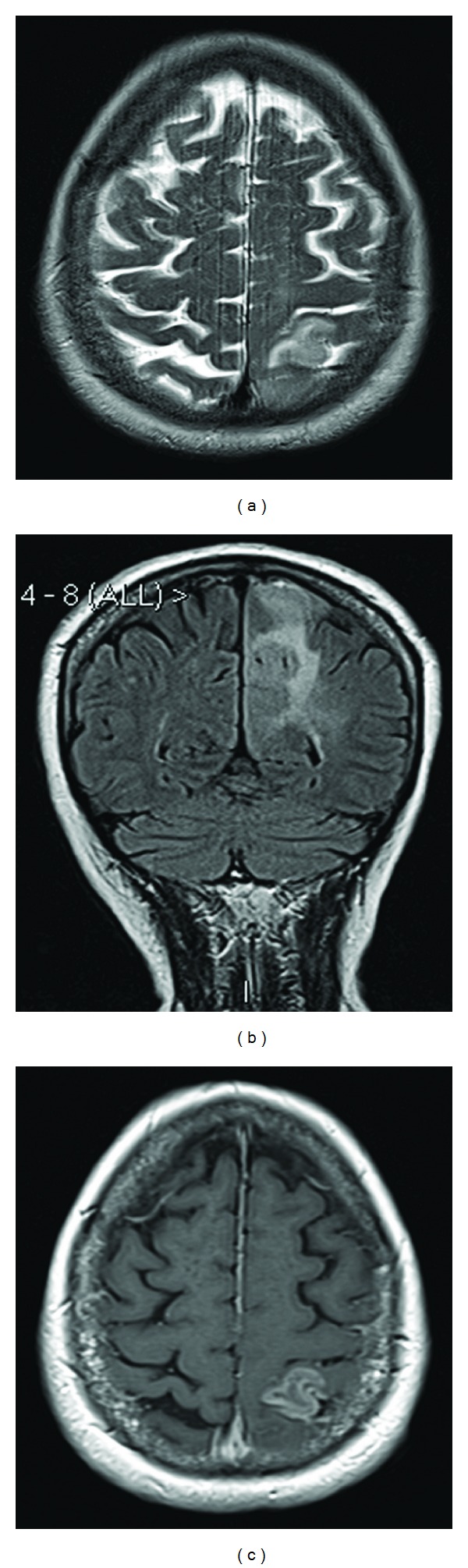
Axial T2 (a), coronal FLAIR (b), and axial T1 post-Gadolinium image (c) from 6-months followup MR scan, showing significant progression of the lesion and a new area of enhancement in the left parietal lobe.

The patient presented six months later with 2 weeks history of partial seizures, involving the right upper and lower limbs. Repeat cranial MRI scan revealed significant progression of the lesion into the occipital region and an increase in the mass effect. An area of enhancement in the left parietal lobe was also present, measuring 19 × 18 mm (Figure 4). The patient subsequently went on to have a biopsy. The pathology confirmed the presence of glioblastoma multiforme (GBM).

## 3. Discussion

Established MRS applications in cerebral tumours include differentiation between brain abscess and cystic tumours, low-grade and high-grade gliomas, gliomatosis cerebri, oedema and tumour infiltration, recurrent tumour, and radionecrosis and used in a stereotactic biopsy planning of brain lesions [1]. MRS literature in brain tumours is based on the assumption that MRS is highly sensitive technique and is highly capable of finding abnormal metabolic spectra in tumours and normal tissues, and questions are usually raised regarding its specificity. We found hardly any studies that raise questions on the inability of MRS to find abnormal metabolites in abnormal looking lesions on MRI. There were no definite false negative rates of MRS reported. There are, however, some controversies in relation to MRS in gliomatosis cerebri.

The most frequently assessed chemical ratios in diagnosis of intracranial tumours is Choline/Creatine (Cho/Cr), Choline/N-acetyl aspartate (Cho/NAA), and Lactate/Creatine (Lac/Cr) ratios. As compared to the normal brain tissue, tumours characteristically present a decreased NAA and increased Cho levels. NAA is normally present in neurons and neuronal injury and replacement of neurons by tumour cells cause reduction in NAA levels. Increased Cho levels are due to breakdown of membrane phospholipids caused by a rapid cell turnover in neoplastic lesions. The usual finding in neoplastic lesions is a Cho/NAA ratio greater than one [1]. The Cho/Cr and Cho/NAA ratios usually vary according to grades of gliomas [2]. 

It is widely believed that adding MRS to conventional MRI protocols improves accuracy of diagnosis of intra-axial tumours. In 2004, Lukas et al. reported the accuracy increased to 94% using long echo time (TE) and to 96% using short TE [3, 4]. Galanaud et al. [5] proposed a diagnostic flow chart for multimodal analysis of brain tumours, that included MR and MRS findings, which correctly identified 91% of intracranial contrast enhancing lesions and 87% of noncontrast enhancing lesions, proving that the addition of MRS to conventional MRI increases significantly the proportion of correct diagnoses of brain tumours. A study by Preul et al. using a pattern-recognition analysis of biochemical information obtained from MRS correctly classified 104 of 105 spectra of five most common types of adult supratentorial brain tumours and a normal brain tissue [6]. These studies largely emphasise the high sensitivity of MRS in brain tumours.

Due to high sensitivity of MRS, it has also been shown to be able to differentiate benign from malignant conditions. Vuori et al. proved that loss of NAA and increase of Cho were more pronounced in low-grade gliomas than in focal cortical developmental malformations [7]. 

The value of MRS in gliomatosis cerebri is more controversial. MRI findings have been reported to be nonspecific and can underestimate the extent of the lesion [8]. The differential diagnosis includes ischaemia, multiple sclerosis, encephalitis, leucodystrophies, and subacute sclerosing panencephalitis [9, 10]. Gliomatosis is believed to be distinct from other gliomas due to the absence of infiltration and preservation of neuronal structures. Overall, there have been limited studies of gliomatosis cerebri with MRS. MRS has been shown to be helpful in detecting abnormal metabolites, thus helping in the diagnosis. In keeping with its high sensitivity, MRS has been used to assess the extent of neoplastic infiltration more accurately than MRI. In addition, MRS has also been shown to be helpful in grading of these lesions [11]. However, there is no consensus about the associated characteristic metabolic abnormalities found in this condition. Some studies report normal choline peaks [11, 12], while others described moderate increase in this metabolite in gliomatosis cerebri [13, 14]. However, the drop in NAA peak is a more consistent described feature [15]. However, we have not come across any reports documenting MRS findings within normal limits even in gliomatosis cerebri.

We have no clear explanation why MRS was normal in our patient. However, given the paucity of reported MRS findings in gliomatosis cerebri in the literature compared to other gliomas, we wonder if it can be to the extent of “within normal limits” as in our patient. Our findings raise questions about the sensitivity and negative predictive value of MRS in gliomatosis cerebri and its ability to differentiate gliomatosis cerebri from other benign cerebral conditions. We do not have histology results at the time of MRS and no MRS data at the time of biopsy, as these were 6 months apart. However, it is likely that the abnormality represented a low grade glioma that dedifferentiated into high-grade glioma a few months later. Hence, it can be reasonably assumed that the MRS data represents low-grade glioma that lasted for a while and was seen as abnormal appearance on MRI, but the MRS findings have been unhelpful in predicting how soon this may occur.

## 4. Conclusion

While it is widely believed that MRS is highly sensitive in gliomas, the current case illustrates that MRS may show normal spectrum in a low-grade glioma or gliomatosis cerebri that can potentially change into a GBM in just a few months time. Therefore, a normal spectrum should not reassure clinicians into assuming a benign aetiology and patients should still be followed up at usual intervals with equal emphasis on other imaging and clinical findings.

## References

[1] Callot V, Galanaud D, Le Fur Y, Confort-Gouny S, Ranjeva J-P, Cozzone PJ (2008). ^1^H MR spectroscopy of human brain tumours: a practical approach. *European Journal of Radiology*.

[2] Moller-Hartmann W, Herminghaus S, Krings T (2002). Clinical application of proton magnetic resonance spectroscopy in the diagnosis of intracranial mass lesions. *Neuroradiology*.

[3] Devos A, Lukas L, Suykens JA, Vanhamme L, Tate AR, Howe FA (2004). Classification of brain tumours using short echo time ^1^H MR spectra. *Journal of Magnetic Resonance*.

[4] Lukas LS, Devos A, Suykens JA (2004). Brain tumour classification based on long echo proton MRS signals. *Artificial Intelligence in Medicine*.

[5] Galanaud D, Nicoli F, Chinot O (2006). Noninvasive diagnostic assessment of brain tumors using combined in vivo MR imaging and spectroscopy. *Magnetic Resonance in Medicine*.

[6] Preul MC, Caramanos Z, Collins DL (1996). Accurate, noninvasive diagnosis of human brain tumors by using proton magnetic resonance spectroscopy. *Nature Medicine*.

[7] Vuori K, Kankaanranta L, Hakkinen A (2004). Low-grade gliomas and focal cortical developmental malformations: differentiation with proton MR spectroscopy. *Radiology*.

[8] Burger PC, Scheithauer BW (1994). *Atlas of Tumour Pathology: Tumours of the Central Nervous System*.

[9] Felsberg GJ, Silver SA, Brown MT, Tien RD (1994). Gliomatosis cerebri: radiologic pathologic correlation. *American Journal of Neuroradiology*.

[10] Shin YM, Chang KH, Han MH, Myung NH, Chi JG, Cha SH (1993). Gliomatosis cerebri: comparison of MR and CT features. *American Journal of Roentgenology*.

[11] Bendszus M, Warmuth-Metz M, Klein R (2000). MR spectroscopy in gliomatosis cerebri. *American Journal of Neuroradiology*.

[12] Galanaud D, Chinot O, Nicoli F (2003). Use of proton magnetic resonance spectroscopy of the brain to differentiate gliomatosis cerebri from low-grade glioma. *Journal of Neurosurgery*.

[13] Mohana-Borges AVR, Imbesi SG, Dietrich R, Alksne J, Amjadi DK (2004). Role of proton magnetic resonance spectroscopy in the diagnosis of gliomatosis cerebri: a unique pattern of normal choline but elevated myo-inositol metabolite levels. *Journal of Computer Assisted Tomography*.

[14] Pyhtinen J (2000). Proton MR spectroscopy in gliomatosis cerebri. *Neuroradiology*.

[15] Guzmán-de-Villoria JA, Sánchez-González J, Muñoz L (2007). ^1^H MR spectroscopy in the assessment of gliomatosis cerebri. *American Journal of Roentgenology*.

